# Feasibility, Acceptability, and Impact of a Web-Based Structured Education Program for Type 2 Diabetes: Real-World Study

**DOI:** 10.2196/15744

**Published:** 2020-01-06

**Authors:** Shoba Poduval, Louise Marston, Fiona Hamilton, Fiona Stevenson, Elizabeth Murray

**Affiliations:** 1 Research Department of Primary Care and Population Health University College London London United Kingdom; 2 University College London London United Kingdom

**Keywords:** diabetes mellitus, type 2, self-management, patient education, internet, digital divide, social class, health literacy, computer literacy

## Abstract

**Background:**

Structured education for people with type 2 diabetes improves outcomes, but uptake is low globally. In the United Kingdom in 2016, only 8.3% of people who were referred to education programs attended the program. We have developed a Web-based structured education program named *Healthy Living for People with type 2 Diabetes* (HeLP-Diabetes): *Starting Out* (HDSO), as an alternative to face-to-face courses. A Web-based program gives people more options for accessing structured education and may help improve overall uptake.

**Objective:**

The aim was to explore the feasibility and acceptability of delivering a Web-based structured education program (named *HeLP-Diabetes: Starting Out*) in routine primary health care and its potential impact on self-efficacy and diabetes-related distress.

**Methods:**

HDSO was delivered as part of routine diabetes services in primary health care in the United Kingdom, having been commissioned by local Clinical Commissioning Groups. Quantitative data were collected on uptake, use of the program, demographic characteristics, self-reported self-efficacy, and diabetes-related distress. A subsample of people with type 2 diabetes and health care professionals were interviewed about acceptability of the program.

**Results:**

It was feasible to deliver the program, but completion rates were low: of 791 people with type 2 diabetes registered, only 74 (9.0%) completed it. Completers improved their self-efficacy (change in median score 2.5, *P*=.001) and diabetes-related distress (change in median score 6.0, *P*=.001). Interview data suggested that the course was acceptable, and that uptake and completion may be related to nonprioritization of structured education.

**Conclusions:**

The study provides evidence of the feasibility and acceptability of a Web-based structured education. However, uptake and completion rates were low, limiting potential population impact. Further research is needed to improve completion rates, and to determine the relative effectiveness of Web-based versus face-to-face education.

## Introduction

### Background

Diabetes self-management education (DSME) aims to help people develop the knowledge and skills to manage their physical and emotional health [1]. There is evidence that DSME can improve glucose control [2,3] and prevent complications [4].

The National Diabetes Audit data in the United Kingdom indicates that only 8.3% of people who were referred to structured education attended in 2016 [5]. Established programs involve face-to-face group courses. Qualitative studies suggest that some people find the face-to-face courses difficult to attend because of timings of courses, lack of transport, work and family commitments, or they do not like taking part in groups [6]. Computer-based self-management interventions bypass some of the barriers to face-to-face education [7,8]. A 2013 Cochrane systematic review of computer-based diabetes self-management interventions found a small effect on glycemic control, which was larger in the mobile phone app subgroup [7]. A 2015 systematic review of internet-delivered DSME found significant improvements in glycemic control and clinic attendance compared with usual care [8]. Some studies in the review also found improvements in self-efficacy [9], diabetes knowledge [10-13], exercise behaviors [14,15], and self-care behaviors [14,16]. A 2017 systematic review of the reviews of technology-enabled diabetes self-management interventions found that 18 of 25 reviews reported a significant reduction in glycated hemoglobin; however, a meta-analysis was not conducted because of heterogeneity in interventions and study designs [17]. Reviews of Web-based diabetes self-management interventions are promising, but there are also challenges including low uptake and engagement, that can limit effectiveness and need further research [18].

In light of this, we developed a Web-based structured education program for people with type 2 diabetes mellitus (T2DM), called *Healthy Living for People with type 2 Diabetes* (HeLP-Diabetes): *Starting Out* (HDSO). The content and aims of the HDSO program were based on an earlier intervention named HeLP-Diabetes. The key difference between HeLP-Diabetes and HDSO was that HDSO was aimed at people who were newly diagnosed, and so it followed a structured written curriculum with specific aims and objectives, as recommended by the National Institute for Health and Care Excellence [19] and required for general practitioners (GPs) to receive quality and outcomes Framework remuneration for referral [20]. HeLP-Diabetes was developed for people at any stage of their illness, and contained information on 560 webpages, which people could dip in and out of without following a linear pathway. A randomized controlled trial (RCT) showed HeLP-Diabetes to be effective and cost-effective [21,22].

The HDSO program is discussed in this paper. HDSO was a Web-based intervention developed for use on desktop computers and tablets. The program development took an iterative user-driven approach informed by the human-computer interaction (HCI) design lifecycle [23,24] and the Medical Research Council (MRC) guidance on developing and evaluating complex interventions. The development process consisted of 3 phases: (1) initial design; (2) usability testing with volunteers; and (3) *in the wild* testing in the National Health Service (NHS) with people newly diagnosed with type 2 diabetes. The 3 stages of the development process are described in detail elsewhere [25].

In line with the MRC framework on the development and evaluation of complex interventions, and current advice on development and evaluation of digital health interventions [26,27], following the development process, we undertook a formative evaluation of the program to explore its feasibility, acceptability, and apparent impact on users. As this was a digital health intervention, it was appropriate to draw on methods more familiar to computer science and HCI researchers than biomedical ones, including an emphasis on *real-world* data, with participants using the intervention as they would in routine practice, rather than as part of an overt research project. Such *real-world* studies, also known as *in the wild* studies, provide a *contextual backdrop* for determining the strengths and weaknesses of the intervention accurately [28]. This allows digital health interventions to be tested by *representative users attempting representative tasks in representative environments* and makes any recommendations for further research user-led rather than researcher-led [29]. Studies *in the wild* can reveal complex and unexplained phenomena that can only emerge in the natural setting of the intervention [30]. They benefit from the strong external validity achieved by delivering the intervention as it will be used in routine practice, and not as part of an overt research project. *In the wild* studies are a necessary precursor to RCTs to determine effectiveness and cost-effectiveness as they allow for further refinement and optimization of the intervention, including the surrounding delivery package provided by health care professionals (HCPs), and a preliminary estimation of any associated changes in outcomes associated with the use of the intervention [27]. Such studies are not intended to, and cannot, determine any causal relationship between observed changes and the intervention tested.

In this study, we took advantage of the naturalistic, *real-world* setting and data provided by 5 Clinical Commissioning Groups (CCGs) commissioning HDSO in the NHS in the United Kingdom, as part of an overall menu of DSME offered to people with T2DM.

### Objectives

The overall aim of the study was to determine the feasibility, acceptability, and potential impact of delivering a Web-based structured education program (HDSO) in routine primary health care. Specific objectives were as follows:

1. Describe people’s use of the program, including numbers (proportions) registering, starting, and completing the program.
2. Determine the demographic, clinical, and psychological factors associated with completion of the program.
3. Investigate the impact of the program on users’ levels of diabetes-related distress and diabetes self-management self-efficacy (DSMSE).
4. Explore the views of people with T2DM and health professionals about the program, including reasons for engagement or nonengagement.

## Methods

This was a mixed method study with a strong emphasis on *real-world* data and external validity. Quantitative data included the proportion of people starting and completing the program, and diabetes-related distress and diabetes self-management self-efficacy scale (DSMSES) questionnaire scores. Interviews with program users and HCPs provided further insights.

### Setting

The study was conducted during implementation of the program in the NHS. People with T2DM in GP practices in 5 London CCGs registered for the program as an NHS service, rather than as research participants. This meant that we were unable to randomize participants to an intervention or control group, or collect clinical outcome data as we did not have access to their clinical records. However, this meant that the participants more accurately reflected the population of interest (people with type 2 diabetes treated in NHS primary health care) [31]. The total population of the 5 CCGs was 1,384,000. The population was diverse, with over 30% of the population from black and minority ethnic (BAME) groups [32]. All 5 CCGs were in the top quartile for deprivation in England [33]. RCTs are more limited in their external validity because of the characteristics of people who volunteer, and the inclusion and exclusion criteria in trial protocols [34,35].

### Ethical Approval and Consent to Participate

Ethical approval was granted by the Health Research Authority (HRA; reference number 159488). The program was offered to people as part of service delivery, so the use of the data on registrations, activities, and questionnaire scores generated through the Web-based program was permissible under the HRA clause that the secondary use of information collected in the course of normal care is generally excluded from the Research Ethics Committee review [36] and specific informed consent to participate in research is not required. However, everyone who volunteered to be interviewed provided full informed consent before being interviewed and was aware that the interviews were for research purposes.

### Participants for the Intervention

The target population was adults with T2DM. Referral was not limited to people who were newly diagnosed, as many people only become ready for structured education after having come to terms with the diagnosis [37-39]. Referral therefore included people at any stage of diabetes to enhance uptake. As this was a service, there were no formal exclusion criteria, but we advised HCPs that people who could not use a computer because of severe mental or physical impairment, had insufficient mastery of English, or were currently participating in a trial of an alternative self-management program would not be suitable for referral. A sample size calculation, which would be used to determine a statistically significant treatment effect in RCTs, was not used as this was not appropriate for the study design.

### Participants for Interviews

Everyone who registered for the program was invited to take part in interviews. We also invited HCPs working at practices who referred a high or low number of people with T2DM, and staff employed by the CCGs to support implementation of the program.

### Recruitment

The program was offered to people with T2DM using referral packs sent by practices who identified eligible people from electronic medical records searches, text messages, flyers, or in consultations with doctors or nurses. In 1 CCG group, there was a dedicated Change Manager who visited practices to talk to staff and people with T2DM about the program. Data on the number of people who were offered the program and declined were not recorded by practices.

People with T2DM were asked to telephone or email the HDSO team, to be registered, and have baseline demographic and clinical data collected (see Outcome Measures). A username and password was emailed to users, along with contact information in case of problems. However, this process of telephone registration proved to be time consuming and caused delay in people being able to access the program. The registration process was therefore modified (see Multimedia Appendix 1) so that users had the option of Web-based self-registration. People with T2DM were given the registration webpage details at referral by HCPs and in referral packs, which they could access to enter their demographic details, and register a username and password to use to log into the program.

### Consent Procedures

#### Quantitative Data (Collected as Part of Service Evaluation)

Users were informed that anonymized data on their use of the program and questionnaire scores were automatically collected by the website for service development. Users were asked to email the team if they did not wish their data to be used. Data were automatically pseudoanonymized and stored locally on a secure network drive. The data were password protected and were only made available to appropriate members of the research team, in keeping with University College London data protection regulations.

#### Qualitative Data

People with T2DM and HCPs who expressed interest in the interviews at registration were contacted by the lead researcher (SP) and given written information about the study and the opportunity to ask questions. After informed consent was received, audiorecorded interviews were undertaken via telephone or in person. Consent forms were stored securely and separately from questionnaire data and audio recordings.

### Intervention

The intervention was a Web-based structured self-management program for people newly diagnosed with T2DM. It is described in detail in Multimedia Appendix 1, using the Template for Intervention Description and Replication (TIDier) checklist [40], and summarized here.

The content was based on HeLP-Diabetes [41], an online self-management intervention for everyone with T2DM. The theoretical basis was the Corbin and Strauss theory that self-management involves 3 tasks: managing the disease process, managing the emotional consequences, and managing the changes that occur in daily life [42]. HDSO was a 4-session program, comprising 4 or 5 modules per session, and questionnaires measuring diabetes-related distress and diabetes self-management self-efficacy score (DSMSES) in weeks 1 and 4. Each module took about 15 to 20 min to complete (see Table 1). Information was presented using text, images, and videos (see Multimedia Appendix 2). Email reminders were sent to users if they had not logged on for 7 days or more.

**Table 1:** *Healthy Living for People with type 2 Diabetes: Starting Out* program sessions and modules.

Session title	Module title
Week 1: Getting started	Module 1: An introduction to type 2 diabetes Module 2: Self-assessment Module 3: Eating well for diabetes Module 4: Becoming more active
Week 2: Self-management	Module 1: Taking control Module 2: Protecting my body and mind Module 3: Handling my feelings Module 4: Making changes (including *My goals and plans*)
Week 3: Improving my health and well-being	Module 1: Making the most of the National Health Service Module 2: Medication Module 3: Reducing the risks of heart attacks and stroke Module 4: Updating my goals and plans Module 5: Understanding my moods
Week 4: Taking control of my diabetes	Module 1: My diabetes review Module 2: Looking after my feet Module 3: Reviewing my goals and plans Module 4: Self-assessment Module 5: Moving on—the beginning of the end

Users were provided with their scores from the diabetes-related distress and DSMSES questionnaires, and individualized feedback developed by GPs and Diabetes Specialist Nurses (DSNs). The feedback in week 1 helped users identify gaps in knowledge or skills, and signposted to sessions of the program that could help them improve. The feedback in week 4 focused on improvements made and directed users to the HeLP-Diabetes website for ongoing support. Users were asked to set specific, measurable, achievable, realistic, time-bounded goals [43], and they were given opportunities to review their goals.

### Outcome Measures

The data collected reflect the objectives of the study (see Table 2).

The server side of the website automatically collected anonymized data on user ID, date and time of login, and pages visited. These were used to determine how many people registered for and completed the program.

Data on personal characteristics included the following: (1) age; (2) gender; (3) ethnicity; (4) highest educational attainment; (5) internet access (home or public); (6) information technology skill level (basic, intermediate, or advanced; (7) duration of diabetes; (8) previously offered face-to-face education (yes or no); (9) previously attended face-to-face education (yes or no); and (10) diabetes management (lifestyle alone, or tablets and/or insulin). Education level was categorized using UK and US qualifications, and the International Standard Classification of Education [44].

Data on change in diabetes-related distress and self-efficacy in self-management were collected using the Problem Areas in Diabetes (PAID) [45] and DSMSES [46] questionnaires.

**Table 2:** Outcome measures.

Objective	Measure	How and when collected
People’s use of the program, including numbers (proportions) registering, starting, and completing the program	Number of people with T2DM^a^ who registered with the program, started the program, and completed it	Data collected on the server side of the website throughout the study and analyzed at the end of the study
Characteristics of people with T2DM registering for the program	Age Gender Ethnicity Highest educational attainment Information technology skill level (basic, intermediate, or advanced) Duration of diabetes (<1 year/>1year) Offered face-to-face education (yes or no) Attended face-to-face education (yes or no) Diabetes management (lifestyle alone or tablets and/or insulin)	Collected over the telephone by the HeLP program team, or using a Web-based questionnaire at registration
Effect of the program on diabetes-related distress and DSMSES^b^	Change in Problem Areas in Diabetes and DSMSES questionnaire scores	Questionnaires completed online by users at baseline (week 1 of the program) and follow-up (week 4 of the program)
View of people with T2DM and health professionals about the program, including factors affecting acceptability of the program	Qualitative interview data from interviews with people with T2DM and HCPs^c^	Interviews conducted after quantitative data collected

^a^T2DM: type 2 diabetes mellitus.

^b^DSMSES: diabetes self-management self-efficacy scale.

^c^HCP: health care professional.

#### Problem Areas in Diabetes

Diabetes-related distress was chosen as an outcome measure as there is a strong correlation between diabetes-related distress and diabetes self-care behaviors, and strong predictive validity for glycemic control [47]. Furthermore, at least 4 in 10 people with diabetes experience diabetes-related emotional distress [48], and addressing emotional distress should be part of comprehensive care for everyone with T2DM [49]. PAID has 20 items on areas that can cause difficulty including social situations and social support [50]. Scores range from 0 to 100, with higher scores indicating more distress. A score of more than 40 is clinically significant [45]. The PAID questionnaire has been shown to have high reliability and validity [51].

#### Diabetes Self-Management Self-Efficacy

Perceived self-efficacy is an individual’s perception of their ability to undertake a task [52]. Diabetes requires a high level of self-efficacy because of the high number of self-management tasks required to prevent complications [46]. Perceived self-efficacy is a reliable predictor of initiation of healthy lifestyle behaviors [53,54]. The 20-item DSMSES questionnaire measures the individual’s expectations of being able to engage in self-management activities such as keeping to a healthy eating plan when away from home [46]. A self-efficacy score of 0 indicates no self-efficacy and a score of 150 indicates very high self-efficacy. The DSMSES questionnaire has strong face validity, and it is a reliable scale for measuring self-efficacy in diabetes research [46].

A total of 13 interviews were carried out by SP (a female academic GP) and 4 interviews were conducted by RB (a male HCI specialist). The interviews lasted between 30 and 60 min. Both interviewers used a topic guide containing questions addressing the aim of the study, including the experience of diagnosis and information seeking; registering for the program; factors that may have affected engagement with the program such as problems using the website; barriers to starting or working through the program; and features or content of the program they liked or disliked. The topic guide for HCPs was tailored for the different roles the staff had in promoting and referring to the program, for example, GPs and nurses were asked about their experiences of discussing HDSO during clinical encounters with people with T2DM, while professionals in nonclinical roles were asked about how they supported clinicians with referring people to the program. Despite using the same topic guide, the different professional roles and interviewing techniques of the 2 interviewers may have led to different responses from the people who were interviewed.

### Analysis

#### Quantitative

Data on the number of people registered for the program and the pages they visited were used to calculate the proportions of people registered for the program who started and completed it. Web-based questionnaire scores were analyzed, and as they were not normally distributed, median and lower and upper quartiles of the scores were calculated. Nonparametric Wilcoxon signed-rank tests were used to determine whether there were any significant differences between the start (week 1) and end (week 4) of the program.

Characteristics of completers and noncompleters of the program were compared using Chi-square tests (or Fisher exact tests where appropriate) to see if there were any factors associated with completion. Mean baseline questionnaire scores of completers and noncompleters were compared using *t* tests. Statistical analyses were carried out using SPSS Statistics version 22 (IBM).

#### Qualitative

Interviews were recorded on a digital audiorecorder and transcribed verbatim by a professional transcription company. Transcripts were anonymized and checked for accuracy by the lead author (SP). Interview findings were analyzed thematically to explore the perspectives of people with T2DM and HCPs, with a particular focus on exploring similarities and differences between perspectives of professionals and people with T2DM [55,56].

Data were analyzed using the following steps: (1) The transcripts were read and re-read by the lead author to allow familiarization with the overall content; (2) the lead author then re-read the transcripts line by line looking for initial codes (text which was relevant to the research question). Codes were highlighted in MS Word; (3) once the lead author was confident that all the data relevant to the research question had been coded, these initial codes were copied and pasted into a separate document. Codes were compared with look for similarities and differences. Similar codes were grouped into potential themes; (4) to maximize rigor, transcripts, codes, and themes were also read by and discussed with FS, EM, and qualitative researchers attending 2 data clinics. The data clinics comprised exploration of the interpretations of the data, and consideration of refinements to existing themes and generation of new themes with 6 qualitative researchers from a range of disciplines (including health services research, sociology, and psychology). After these discussions with colleagues, the transcripts were re-read by the lead author (SP) who refined the themes and then discussed them with FS and EM until consensus was reached.

#### Applying Concepts of Normalization Process Theory to the Data

Once the themes were agreed upon, it became clear that some of the themes related closely to the constructs from Normalization Process Theory (NPT). NPT is concerned with making new interventions routine practices in health care (embedding) and sustaining embedded practices (integration) [57]. The theory operationalizes the work of implementation as 4 constructs: (1) coherence (sense making of the intervention); (2) cognitive participation (commitment of the participant); (3) collective action (the work participants do to make the intervention function); and (4) reflexive monitoring (how participants appraise the intervention). We used knowledge of the NPT constructs, the intervention, and the intervention setting to define intervention-specific meanings for each construct. We then mapped these constructs to the appropriate themes from the interviews. This 2-stage process of analysis has been used in other qualitative studies of complex interventions in primary health care [58] and provides a deeper analysis, by allowing researchers to embed the findings in existing theoretical concepts and in this way provide a theoretically informed interpretation in relation to implementation.

## Results

### Use of the Program

A total of 791 people registered for the program, 188 started it (completed at least the first module of the first session), and 74 completed all 4 sessions (see Multimedia Appendix 3).

### Characteristics of Participants

Data on the characteristics of people with T2DM is given in Table 3. A total of 791 people with T2DM registered to use the program. Demographic data were self-reported at registration (by either telephone or Web-based questionnaire), and there is a large amount of missing data because of nondisclosure, particularly on previous offer and attendance at structured education and diabetes management. The average age of people with T2DM registering to use the program was 57.6 years, over half (316/586, 53.9%) were male, over half (310/605, 51.2%) were from BAME backgrounds, and nearly one-third (181/602, 30.1%) had no qualifications beyond high school leaving age (Table 3). Just over one-quarter (170/589, 28.9%) had had their diabetes <1 year, and while half (193/394, 49.0%) recalled being offered face-to-face education, only 9.4% (37/394) had attended it.

### Characteristics of Completers

The only factors associated with completion were duration of diabetes (*P*=.04), and having been offered (*P*=.001) and having previously attended (*P*=.002) face-to-face education. Having advanced information technology skills was not associated with completing the program (see Table 3).

**Table 3:** Characteristics of registered people with type 2 diabetes mellitus (T2DM), completers, and noncompleters.

Variable		Registered people with T2DM		Completers		Noncompleters		*P* value
		Mean (SD) or n (%)	N	Mean (SD) or n (%)	N	Mean (SD) or n (%)	N	
Age (years)		57.6 (12.9)^a^	749	56.7 (13)^a^	749	56.8 (20.9)^a^	749	.63
Sex (male)		316 (53.9)	586	36 (51)	71	280 (54.4)	515	.50
**Ethnicity**								
	White	287 (47.4)	605	37 (51)	72	250 (46.9)	533	.53
	Black	206 (34.0)	605	24 (33)	72	182 (34.1)	533	N/A^b^
	Asian	67 (11.1)	605	8 (11)	72	59(11.1)	533	N/A
	Mixed	20 (3.3)	605	2 (3)	72	18 (3.4)	533	N/A
	Other	17 (2.8)	605	0 (0)	72	17 (3.2)	533	N/A
	Prefer not to say	8 (1.3)	605	1 (1.4)	72	7 (1.3)	533	N/A
GCSE^c^/high school diploma		181 (30.1)	602	19 (28.4)	67	162 (32.9)	492	.45
Basic or intermediate information technology skills		406 (71.5)	568	46 (71)	65	360 (71.6)	503	.89
Diabetes duration <1 year		170 (28.9)	589	32 (45.1)	71	138 (28.5)	485	.04
Offered face-to-face education		193 (49.0)	394	42 (69)	61	151 (45.3)	333	.001
Attended face-to-face diabetes education		37 (9.4)	394	11 (18)	62	26 (7.8)	333	.002
Lifestyle alone (ie, diet and physical activity)		111 (28.2)	394	19 (31)	62	92 (27.7)	332	.22
Tablets and/or insulin		283 (71.8)	394	43 (69)	62	240 (72.3)	332	N/A

^a^Refers to mean (SD).

^b^N/A: not applicable.

^c^GCSE: general certificate of secondary education.

### Impact of Completing the Program

Median DSMSES (self-efficacy) scores were significantly higher (better) in week 4 compared with week 1. Median PAID (distress) scores were significantly lower (better) in week 4 than week 1 (see Table 4).

**Table 4:** Baseline and follow-up questionnaire scores.

Questionnaire	Week 1 median (LQ^a^,UQ^b^)	Week 4 median (LQ, UQ)	*P* value
PAID^c^	7.50 (4.00, 11.25)	5.00 (2.00, 9.00)	.001
DSMSES^d^	101.50 (78.00, 119.25)	107.50 (95.50, 130.50)	.001

^a^LQ: lower quartile.

^b^UQ: upper quartile.

^c^PAID: problem areas in diabetes.

^d^DSMSES: diabetes self-management self-efficacy scale.

### Views of People With T2DM and Health Professionals About the Program

Interviews were conducted with 17 participants (10 people with T2DM and 7 HCPs). Of the 10 people with T2DM, 7 had completed the program and 3 had registered for the program, but not completed it. Other characteristics are listed in Multimedia Appendix 4. Of the HCPs, 3 were DSNs, 2 were GPs, 1 was a HeLP-Diabetes Change Manager (employed by the local CCG to liaise with the GP Practices and promote the HDSO program) and 1 was a CCG Project Officer (providing support to senior CCG project managers). The data from people with T2DM and HCPs are combined in the results, as many of the subthemes are shared. Where a subtheme is unique to a particular group, this is stated in its description and illustrated with a quote.

Four major themes emerged from the analysis of the interview data, each with a number of subthemes (see Table 5). We mapped 2 of the major themes to NPT constructs. NPT explains whether and how complex interventions become embedded in health care practice. Hence, the themes that mapped to the constructs were those that related to health care system factors (the value of discussion between HCPs and people with T2DM about DSME at the time of referral; and improving uptake of the HDSO program) rather than factors that related to people with T2DM or the program. The NPT constructs and the HDSO-specific meanings we defined for each construct are listed in Table 6. The themes and subthemes we mapped to the constructs are listed in Table 7.

**Table 5:** Major themes and subthemes from the qualitative data.

Major theme	Subthemes
Lack of discussion between HCPs^a^ and people with T2DM^b^ about DSME^c^ at the time of referral	Poor understanding of structured education by professionals Lack of time to discuss structured education
Factors affecting people’s motivation toward DSME	Competing priorities Not being ready for information Perceived lack of relevance Perceived lack of need
User experience and advantages of a Web-based education program	Convenience Format Emotional support
Improving uptake of the HDSO^d^ program	Supporting HCPs with referrals Changes to the program

^a^HCP: health care professional.

^b^T2DM: type 2 diabetes mellitus.

^c^DSME: diabetes self-management education.

^d^HDSO: Healthy Living for People with type 2 Diabetes: Starting Out.

**Table 6:** Normalization process theory constructs and *Healthy Living for People with type 2 Diabetes: Starting Out*–specific meanings of the constructs.

Constructs	HDSO^a^-specific meaning of the constructs
1. Coherence (sense-making of the intervention; anchoring in experience)	How well HCPs^b^ understood the HDSO program and how it was different to face-to-face courses. Whether HCPs valued the projected benefits of the HDSO program to people with T2DM^c^ and the primary care team, and whether they developed a shared sense of benefit of the program.
2. Cognitive participation (engagement and commitment of the participant)	The engagement of HCPs in the HDSO program, whether they thought it was a good idea, and whether they were willing to invest time, energy, and work into it.
3. Collective action (the work participants do to make the intervention function)	The additional work for practices of promoting the program (including sending recruitment packs or text messages, and printing and displaying flyers in waiting areas). The work for HCPs of fitting discussions about DSME and referrals to the HDSO program into time-limited consultations. Any additional training needed to be able to explain and demonstrate the program, and send referrals.
4. Reflexive monitoring (how participants reflect on or appraise the intervention)	Whether HCPs perceived the worth of the HDSO program, and its impact on their other tasks.

^a^HDSO: Healthy Living for People with type 2 Diabetes: Starting Out.

^b^HCP: health care professional.

^c^T2DM: type 2 diabetes mellitus.

**Table 7:** Mapping of themes onto normalization process theory constructs.

Major theme and subtheme		NPT^a^ construct
**Major theme 1: Lack of discussion between HCPs^b^ and people with T2DM^c^ about DSME^d^ at the time of referral**		
	Poor understanding of structured education by professionals	Coherence
	Lack of time to discuss structured education	Collective action
**Major theme 4: Improving uptake of the HDSO^e^ program**		
	Familiarizing professionals with the program	Collective action; reflexive monitoring
	Health assistant or administrative assistant-led referral	Collective action

^a^NPT: normalization process theory.

^b^HCP: health care professional.

^c^T2DM: type 2 diabetes mellitus.

^d^DSME: diabetes self-management education.

^e^HDSO: Healthy Living for People with type 2 Diabetes: Starting Out.

#### Lack of Discussion Between Health Care Professionals and People with Type 2 Diabetes Mellitus About Diabetes Self-Management Education at the Time of Referral

HCPs and people with T2DM both expressed a sense of dissatisfaction about the way structured education was discussed at the time of referral. When people with T2DM described how they were informed about structured educations they explained that they received written confirmation of referral to an education program, but no discussion with HCPs about what the education program involves or the benefits of attending.

How much information did you get from your GP and your practice nurse about the diabetes when you first got it?

Interviewer

I don’t remember getting that much, just referrals. I got it in a letter. She didn’t call me and say, you have diabetes, so you have tipped over and we are now referring you.

Participant 4, 63-year-old female noncompleter, duration of illness 1-5 years

#### Poor Understanding of Structured Education by Professionals

People with T2DM did not express views on why discussions about DSME with HCPs were limited, but HCPs identified contributing factors as being lack of time in consultations (collective action) and poor HCP understanding of the nature and benefits of structured education (cognitive participation):

The mode of referral played a part in how effectively people took up structured education programs. And a lot of this is due to, I think there are two main facts. One, lack of knowledge about what structured education is amongst health care professionals, and also the time and type of engagement that people have when engaging with patients who have been newly diagnosed with diabetes.

Participant 14, GP, and Clinical Director

#### Lack of Time to Discuss Structured Education

All the HCPs who were interviewed agreed that there is no opportunity for significant discussion of DSME, as there is not enough time in the consultation to explain or (in the case of the HDSO program) demonstrate the program, as well as manage the person’s other problems:

I think you’re asking the impossible. GPs have a few minutes, the practice nurses probably have 20 minutes, at best...

Participant 17, Diabetes Specialist Nurse

#### Factors Affecting People’s Motivation Toward Diabetes Self-Management Education

We asked both HCPs and people with T2DM about factors which may have contributed to whether people who registered for the program used it or not. We were particularly interested in why people registered for the program but did not complete it. All 10 of the people with T2DM who were interviewed registered for the program. Then, 3 started the program but did not complete it, and 7 started the program and did complete it. Both completers and non-completers described how competing priorities got in the way of having time to work through the program. HCPs and people with T2DM questioned whether people at an earlier stage of their illness might not feel ready for the information in the program or perceive the information as lacking relevance to them.

#### Competing Priorities

People described having other priorities competing for their time when they were working through the program, which meant stopping and starting and having long periods of not using the program at all. Competing priorities included work, as most people with T2DM who were interviewed were working age, and family responsibilities.

And yes, from time to time I got phone calls which were helpful and it just, again, just sort of urge you to get on to the program if you’d had a long gap from going on to doing it, yes.

Participant 5, 64-year-old female completer, duration of illness 1-5 years

So was that something that you found difficult? Because what we try and get people to do, is to do a session a week or a session every two weeks so that it kind of proceeds at a good pace. Did you find that difficult?

Interviewer

No that wasn’t difficult. I think it was again just due to work. It may have fallen over periods where I’m so busy at work that I just didn’t have time to do it.

Participant 5, 64-year-old female completer, duration of illness 1-5 years

#### Not Being Ready for Information

HCPs expressed their concern that people with T2DM at an earlier stage of the illness do not feel the need to take self-management seriously yet. They described how people with T2DM without complications may feel well and have no symptoms, and so are not ready or willing to take on information about changing their lifestyle through self-management.

If we’re talking about complications it’s too far away for them to think about, if we’re talking about behaviour change they think... It’s a disease with no symptoms, largely, and I think that that’s the massive issue, I think that people take it seriously when things start to go wrong.

Participant 17, Diabetes Specialist Nurse

#### Perceived Lack of Relevance

Some people with T2DM saw themselves as *newly diagnosed* even if they had diabetes for more than a year, because they were not yet taking medication to manage the illness. These people talked about how they perceived much of the information in the program as being more relevant to people taking medication and less relevant to them:

A lot of the online (program) also I think targeted people who are on medication so how much as a, sort of a newly diagnosed I don’t know how helpful it was to be honest with you. Because some of it I just felt didn’t apply to me.

Participant 5, 64-year-old female completer, duration of illness 1-5 years

#### Perceived Lack of Need

Some people expressed that they did not feel the need for more information about diabetes self-management, because they believed they already knew what they needed to know, particularly about diet changes:

They probably could give me further hints but at the same time, I just feel I really do know what to do—don’t eat any carbs or sugars or anything and you’ll keep it under control.

Participant 4, 63-year-old female noncompleter, duration of illness 1-5 years

#### User Experience and Advantages of a Web-Based Education Program

Despite the low completion rate, people with T2DM reported enjoying being able to work through the program at their own pace, as opposed to being given a lot of information at one time on a 1-day face-to-face course. People also talked positively about the way information was presented in the program using text, graphics, and videos (particularly videos of others living with the illness). This is crucial in understanding the importance of giving people with T2DM a Web-based option for DSME.

#### Convenience

People with T2DM described a preference for taking their time to work through the large amount of information contained in a self-management education course, as opposed to processing information given in a 1-day face-to-face course:

I think that was the other thing which was really good about this site, is that with the DESMOND [Diabetes Education and Self-Management for Ongoing and Newly Diagnosed], it’s all there in one day, like packed into a day. And... I mean, yes, they... you go away with nice little booklets and that, but I don't... I swear to God; I haven’t really even looked at it. Whereas this, it’s, sort of, like, telling you that you can make tiny little changes.

Participant 1, 61-year-old female completer, duration of illness 1-5 years

#### Format

People with T2DM described enjoying the variety of formats in which the information was presented, including text, graphics, and videos. In particular, people with a preference for learning using visual information appreciated the videos accompanying the text:

The videos explain things... some things really, really well. I think everybody is different, aren’t they? Some people work well with visual stuff, and other people work well with written stuff. I’d always think if you read and look, you know, it gets into the brain. You know, I think those things were really, really good.

Participant 8, 60-year-old female completer, duration of illness less than 1 year

#### Emotional Support

Some people with T2DM found the support provided by the program with managing the emotional side of the illness useful, particularly watching the videos of others talking about living with the illness. This emphasizes the need for structured education programs to acknowledge the emotional challenges of diabetes self-management and the need to include emotional support in courses:

I thought it concentrated a lot about your emotional side. And listening to some of the other people, and I thought, oh, it’s not just me who was annoyed. I know a lot of people got upset, and, I mean, I didn’t get upset, I was just annoyed. And so I felt that there were similar experiences, you know, other people probably, it’s not just me who was feeling that way. The other people reacted probably similar when they found out that they were diabetic.

Participant 1, 61-year-old female completer, duration of illness 1-2 years

#### Improving the Uptake of Healthy Living for People With Type 2 Diabetes: Starting Out Program

The HCPs who were interviewed had a number of suggestions for improving the uptake of the HDSO program. These included supporting professionals to improve the discussion at the time of referral by encouraging them to familiarize themselves with the program before discussion with people with T2DM (collective action and reflexive monitoring); and delegating some of the workload by allowing health care assistants or administrative assistants to refer people to the program (collective action). Other suggestions were made by people with T2DM about changes to the program to provide more personalized information, and making the program available to access on smartphones.

#### Supporting Health Care Professionals With Referrals

As discussed above, all the HCPs expressed that there is not enough time in a clinical consultation to discuss and demonstrate the program to people with T2DM at the time of referral. Suggestions were therefore made about supporting HCPs with referral, including allowing health care assistants and administrative staff to refer people to the program before or after their clinical consultation with a doctor or nurse, when there is more time for discussion:

Ideally, if there were a health care assistant or an admin person who could catch the patient separately either before or after the appointment to show them the website and sign them up, I think that would work really well.

Participant 16, Diabetes Specialist Nurse

#### Changes to the Program

Some people with T2DM expressed the need for more personalized information, particularly specific diet information on what they should and should not be eating. People mentioned that they would like to be able to use the program on their mobile phones, for convenience, and that they were unable to do so with the current format:

I, sort of, get on it and go through it, because I'm in that mood. Then go through two of the modules, let’s say, from part four. I’ve done part four and part five today. But when I wanted to [unclear] on my mobile phone, when I was [unclear], I thought: right, no I’ll sit down on the phone, you know. I jumped on my phone to do the modules. I found it, kind of, difficult. I find I had to restart the modules on my mobile phone. It wasn’t... it wasn’t, sort of... it didn’t jump out at me and I found it quite frustrating.

Participant 7, 47-year-old male completer, duration of illness <1 year

## Discussion

### Principal Findings

This study found that it is feasible to deliver Web-based structured education in the NHS; a wide demographic can use it; and it may improve self-efficacy and diabetes-related distress. The quantitative data showed that there were problems with uptake and completion, with completion positively associated with duration of diabetes <1 year, and self-report of having been offered and/or attended structured education previously. The qualitative data helped us to explore the low uptake more fully. Findings from interviews with people with T2DM and health professionals suggested that professional factors, personal factors for people with T2DM, and program factors affected program acceptability and attrition. More research is needed on improving uptake and determining the relative effectiveness and cost-effectiveness of Web-based and group-based structured education.

### Strengths and Limitations

The strengths of this study include the strong external validity, with our data drawn from *real-world* experience of implementation and delivery of the service within routine primary health care. Other strengths include the use of a mixed method, with quantitative data on uptake, usage, and outcomes, and qualitative data to explore the underlying reasons for these observed data, and the use of theory (NPT) in the data analysis. Using NPT allowed us to make a theoretically informed interpretation of the qualitative data in regard to implementation. The design was appropriate for the study objectives, namely, to determine acceptability, feasibility, and apparent impact of the program. Acceptability and feasibility related not only to patients but also to health professional factors, which NPT allowed to explore more deeply.

It is important to state clearly that this design cannot be used to determine effectiveness of the intervention, nor can any causal links be inferred. Determining effectiveness and ascribing a causal impact of an intervention requires an RCT design, with an appropriate comparator, and a sample size calculation. In light of the multiple tests undertaken, it is possible that the observed associations between likelihood of completion and duration of diabetes, and self-reported offer or attendance at structured education are because of chance. A specific weakness of our study was the lack of clinical outcome data and our reliance on proximal outcomes collected through self-reported outcome measures. This was a direct result of our emphasis on external validity and *real-world* data, so that people with T2DM used the program as part of their routine NHS care, and not as part of a research study. Hence, we could not obtain formal informed consent, except from those who participated in interviews, and as such, it would not have been ethical to have access to clinical data. A further limitation was our inability to invite people who were offered the program but did not register to use it to take part in interviews because of the ethical limitations. Interviewing these people would have helped us to understand why some people do not want to use Web-based structured education.

### Comparison With Previous Work

The percentage of completers was low (9.4%) but compares favorably with attendance at face-to-face education (8.3%) [5] and adherence to other digital self-care interventions [8,59-63]. The interviews helped explore attrition from the program, and NPT improved interpretation and transferability of the themes. Reasons for low engagement were similar to findings from a 2016 systematic review of diabetes education programs [6] and suggest that personal factors for people with T2DM and HCP factors, including nonprioritization, lack of enthusiasm from HCPs for education, and people feeling that they would benefit or that knew enough already, contributed to poor uptake and completion [6].

The study included people with any duration of diabetes, but one of the factors associated with completion was duration of diabetes of less than a year (*P*=.04). The rationale for including people with any duration of diabetes was that the literature suggests that many people only become ready for structured education after having come to terms with the diagnosis [37-39], and we did not want to limit uptake by only including people who were newly diagnosed. Our previous research had shown that uptake was lower when people with diabetes of duration greater than a year were excluded [25]. Overall, our findings therefore suggest that while offering structured education to people with diabetes of any duration does help improve uptake, overall uptake is still low, and it is the newly diagnosed group who are more likely to complete the course. This is consistent with a 2014 study by Roelofson et al, which found low overall participation in a Web-based patient platform for T2DM containing health data and educational information (110 people used the intervention out of 974 who were registered, 11.3%), but interest was higher in people with shorter duration of illness [64]. This suggests that DSME should be offered to everyone with T2DM, but people who are newly diagnosed are a group who should continue to be targeted and offered referral in the first year following diagnosis.

Having been offered face-to-face education (*P*=.001) and having attended face-to-face education (*P*=.002) also seemed to be associated with completion. The association between likelihood of completion and having been previously offered, or attended, structured education has not been reported previously in the literature. This association could reflect intrinsic characteristics, whereby people with more interest in and commitment to structured education are more likely to remember the offer and attend whatever education they are offered, or could reflect an effect of structured education, in that it makes people aware of how much there is to learn, and hence promotes engagement with subsequent offers. Further research is needed on whether people are more likely to take up structured education if they are offered it more than once, or whether more incentives are needed to increase intrinsic motivation and interest and commitment to complete a course.

### Conclusions

If Web-based structured education can be found to be effective and cost-effective and have acceptable reach, this could give people with T2DM more options for learning about self-management and help improve structured education uptake.

## Appendix

Multimedia Appendix 1 Description of Healthy Living for People with type 2 Diabetes: Starting Out according to the Template for Intervention Description and Replication checklist.

Multimedia Appendix 2 Screenshot of Healthy Living for People with type 2 Diabetes: Starting Out.

Multimedia Appendix 3 Flowchart showing numbers of people with type 2 diabetes mellitus registering, starting, and completing the program.

Multimedia Appendix 4 Characteristics of people who participated in interviews.

## Abbreviations

BAME: black and minority ethnic
CCG: Clinical Commissioning Group
DSME: diabetes self-management education
DSMSES: diabetes self-management self-efficacy scale
DSN: diabetes specialist nurse
GP: general practitioner
HCI: human-computer interaction
HCP: health care professional
HDSO: HeLP-Diabetes: Starting Out
HeLP-Diabetes: Healthy Living for people with type 2 Diabetes
HRA: Health Research Authority
MRC: Medical Research Council
NHS: National Health Service
NIHR: National Institute for Health Research
NPT: normalization process theory
PAID: Problem Areas in Diabetes
RCT: randomized controlled trial
T2DM: type 2 diabetes mellitus
TIDier: Template for Intervention Description and Replication
