# Supplementary material for: Feasibility, Acceptability, and Impact of a Web-Based Structured Education Program for Type 2 Diabetes: Real-World Study
Source: JMIR Diabetes. 2020 Jan 6;5(1):e15744. doi: 10.2196/15744 (PMC6971513; doi:10.2196/15744)
Supplement: Multimedia Appendix 1 [file diabetes_v5i1e15744_app1.docx]

**Description of HeLP-Diabetes: Starting Out according to the TIDier checklist**

The TIDier (Template for Intervention Description and Replication) (1) checklist has been used as a guide for information to include in describing the intervention, and the location of information.

1. **BRIEF NAME**
   1. HeLP-Diabetes: Starting Out (HDSO)
   2. An online structured self-management programme for type 2 diabetes.
2. **WHY**
   1. *Rationale* The National Institute for Health and Care Excellence (NICE) recommend that all adults with type 2 diabetes receive structured education at or around the time of diagnosis (2). Uptake is low and National Diabetes Audit data indicates that only 7.5% of referred patients attended in 2015-16 (3). Online structured education programmes bypass some of the barriers presented by face-to-face programmes. HDSO was an online structured education programme which could be used flexibly by patients and could help improve access to structured education.
   2. *Theory* The content of HeLP-Diabetes and HDSO was based on the Corbin and Strauss model of the work of managing a long term condition (4). This is conceptualised as consisting of three tasks: (1) Medical management (adopting healthy behaviours, working with health professionals); (2) Emotional management (addressing the negative emotions associated with being diagnosed with a long-term condition); and (3) Role management (coming to terms with the disruption to one’s sense of self). HDSO took a holistic view of self-management and content addresses a wide range of needs including lifestyle changes, taking medication, interacting with the healthcare system, managing feelings and the impact of the illness on social life, work and relationships.
   3. *Goal* The aim of the HDSO programme was to help people newly diagnosed with type 2 diabetes to improve their knowledge, self-efficacy and emotional wellbeing by learning about living a healthy lifestyle, making the most of the NHS and staying motivated.
3. **WHAT (materials)**

*Components of the intervention*

- - - *Spiral curriculum* The HDSO programme consisted of four weekly sessions, divided into four or five modules. Each module took about 15-20 minutes. The programme could be accessed at [www.help-diabetes.org.uk](http://www.help-diabetes.org.uk). Session headings were: (1) Week 1-Getting started; (2) Week 2-Self-management; (3) Week 3-Improving my health and well-being; and (4) Week 4-Taking control of my diabetes. Each session had a list of learning objectives. Sessions could be saved and returned to later. The programme followed a spiral curriculum which means that people build on the knowledge they have acquired in each session as they proceed.
    - *Self-assessment* Self-assessment questionnaires were included in the programme in Weeks 1 and 4 (pre- and post-programme). These were used to help people recognise their learning needs at the start of the programme, reflect on their learning at the end of the programme, and to provide evidence of impact of the intervention for my research. The questionnaires measure diabetes self-efficacy in self-management (the DSMES questionnaire (5)), and diabetes-related distress (the PAID questionnaire (6)).
    - *Goal-setting* The programme also included goal-setting and action-planning tasks. People were asked to set specific, measurable, achievable, realistic, time-bounded (SMART) goals week 3. They could enter and save their goal onto the website, along with a plan of exactly what they are going to do (e.g. stick to a diet goal but taking a shopping list of low calorie food to the supermarket), potential barriers and ideas for navigating these barriers. People could also select a choice of email or text reminder and a review date, to help them monitor and achieve their goals. In week 4 there was an opportunity to review goals that have been set, and rate progress from 0 to 5. People were asked questions about how they feel about their progress, and given encouragement and feedback such as making a goal more achievable, or setting new reminders.

1. **WHAT (procedures)**
   1. *Registration* The programme was available to adults with type 2 diabetes registered with a practice in one of the five Clinical Commissioning Groups (CCGs) that commissioned the programme. Patients were informed about the programme by health professionals and posters and flyers in waiting areas. Some practices sent adults with type 2 diabetes text messages informing them about the programme. Patients were initially registered by phone, but could later register online by visiting the self-registration page of the website (help-diabetes.org.uk/register). People could contact the HeLP team for support via telephone.
   2. *Personalized emails* Everyone who completed a session received an email with congratulations and an introduction to the next session. Anyone who did not log in for seven days or more received a reminder email explaining that the programme was still available, and introducing the next session. The emails were written by an academic GP (SP) who was a member of the HeLP team.
   3. *Personalized feedback* Everyone who completed the questionnaires in Weeks 1 and 4 received individualized feedback written by GPs and Diabetes Specialist Nurses from the HeLP team. The feedback included the actual score, the total possible score and the lowest scoring topic area. The aim was to give people information on their actual level of knowledge, and the reference level of knowledge. The aim was to help people think about how to move forward with their learning. The emails signposted people to sessions of the course that would help them improve their knowledge and skills. The feedback was written by a GP.
2. **WHO PROVIDED**
   1. *General Practitioners (GPs)* Two academic GPs worked with the HeLP team to implement the HDSO programme in primary care. One had technical expertise and was the IT lead. The other GP was a GP researcher (SP) who led the evaluation and collected data on uptake, questionnaire scores and acceptability to patients and health professionals.
   2. *Diabetes Specialist Nurses (DSNs)* Two DSNs worked with the HeLP team to develop the content and format of the programme. The DSNs also provided advice on the personalised emails and feedback, and visited practices to promote the programme.
3. **HOW (modes of delivery)**

The programme is entirely delivered online, and is developed to be used by individuals. There is no face-to-face contact. People are encouraged to complete one session a week, but can sessions can be saved and returned to later. Email feedback and reminders are sent at various stages of the programme, as described above. Telephone support is available from the HeLP team if people have any problems with the website.

1. **WHERE**

Adults with type 2 diabetes in five CCGs in London were informed about the programme at their GP practices. They were able to register by telephone or self-register for the programme on the website. The programme is completed online, and has been developed for use either on a tablet or a desktop computer. It can therefore be used anywhere where these devices and an internet connection are available.

1. **WHEN and HOW MUCH**

The programme has four sessions which include four or five parts, each taking 15-20 minutes to complete. People were encouraged to complete one session a week but were able to save a session and return later.

1. **TAILORING**

People were sent personalized feedback after they completed the self-assessment questionnaires in Weeks 1 and 4. The feedback included the questionnaire scores, the total possible and score and lowest scoring area. It also signposted to useful parts of the course based on the score. Personalized emails were also sent on completion of a session, introducing the next session that was opened up. Personalized emails were sent to people who did not log in for seven days or more, explaining that the programme was still available and introducing the next part of programme.

1. **MODIFICATIONS**

Self-registration was introduced during the study. This meant that patients who were interested in using the HDSO programme could register themselves by visiting the website’s registration page. This replaced the telephone registration system which relied on the HeLP team calling patients who returned reply slips expressing interest in the programme. No other modifications were made to the programme during the study. The programme had been modified prior to the start of this study, as a result of the outcomes of a previous smaller study. The modifications included reducing the number of self-assessment questionnaires from three to two; reducing the number of sessions in the programme from eight to four; and offering the programme to everyone with type 2 diabetes and not just people who were newly diagnosed (diagnosed in the previous nine months). These changes were made to improve the number of people registering for the programme and completing it.

1. **HOW WELL (planned)**

Registrations for the programme were automatically recorded on the server side of the website. Anonymous user IDs were allocated to everyone who was registered, and the time, date and web page visited every time they logged in. This data were used to calculate the number of registrations, the number of people who completed the programme, and the number of people who completed it. The website also recorded the self-assessment questionnaire scores at Weeks 1 and 4. The anonymised data were downloaded to Excel by a GP researcher, and analysed.

1. **HOW WELL (actual)**

791 patients registered for the program, 188 (23.8%) started it (completed at least one module of one session) and 74 (9.4%) completed it (completed all 4 sessions).

REFERENCES

1. Hoffmann TC, Glasziou PP, Boutron I, Milne R, Perera R, Moher D, et al. Better reporting of interventions: template for intervention description and replication (TIDieR) checklist and guide. BMJ : British Medical Journal. 2014;348.

2. National Institute for Health and Clinical Excellence. Type 2 Diabetes in adults: management. NICE guideline NG28. 2015.

3. Health & Social Care Information Centre. National Diabetes Audit 2013-2014 and 2014-2015 Report 1: Care Processes and Treatment Targets. 2016.

4. Corbin JM, Strauss A. Unending Work and Care. San Francisco: Jossey-Bass Inc; 1988 1988//.

5. Sturt J, Hearnshaw H, Wakelin M. Validity and reliability of the DMSES UK: a measure of self-efficacy for type 2 diabetes self-management. Primary Health Care Research & Development. 2010;11(04):374-81.

6. Welch G, Weinger K, Anderson B, Polonsky WH. Responsiveness of the problem areas in diabetes (PAID) questionnaire. DiabetMed. 2003;20.

7. Doran GT. There's a SMART way to write management's goals and objectives." and Miller. Arthur F & Cunningham, James A" How to avoid costly job mismatches" Management Review. 1981;70(11).
