# Supplementary figures and images for: Feasibility, Acceptability, and Impact of a Web-Based Structured Education Program for Type 2 Diabetes: Real-World Study
Source: JMIR Diabetes. 2020 Jan 6;5(1):e15744. doi: 10.2196/15744 (PMC6971513; doi:10.2196/15744)

## Slide 1
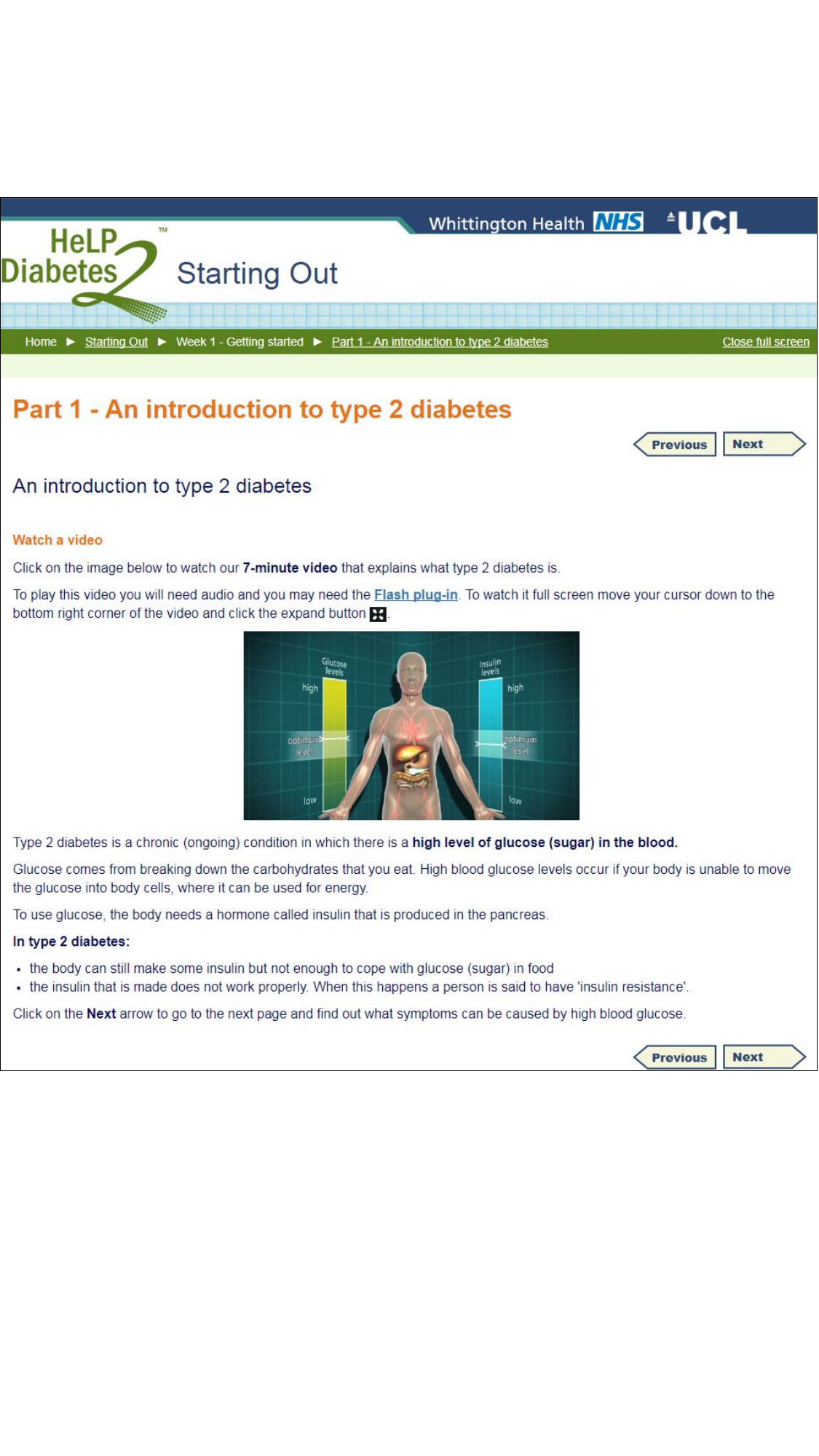

Supplement: Multimedia Appendix 2 [file diabetes_v5i1e15744_app2.pptx]

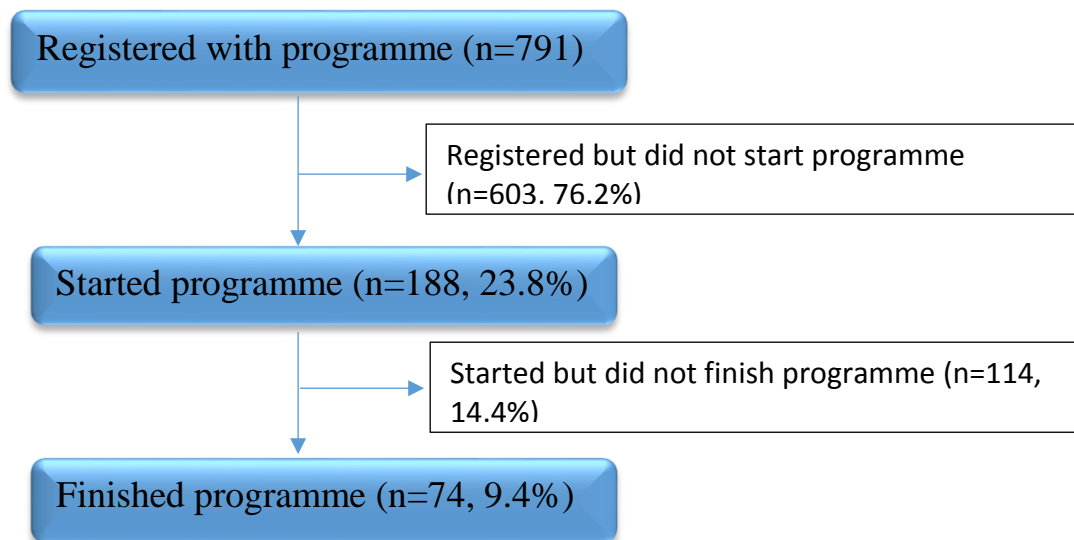

Supplement: Multimedia Appendix 3 [file diabetes_v5i1e15744_app3.pdf]
